# ICF in Bachelor degree programs—the implementation of the ICF in the clinical reasoning process of physical therapists for neurological patients—optimizing the health curriculum for comprehensive patient care

**DOI:** 10.3389/fresc.2024.1412163

**Published:** 2024-08-09

**Authors:** Hannes Aftenberger, Bernhard Taxer

**Affiliations:** Department of Physiotherapy, FH JOANNEUM Graz, Graz, Austria

**Keywords:** physiotherapy, Bachelor degree programs, ICF, clinical reasoning, neurorehabiliation, undergraduate

## Abstract

The International Classification of Function, Disability, and Health (ICF) is known to be a valuable classification model in interprofessional neurorehabilitation, as it can lead to more patient-centered and self-determined treatment. To acquire the competencies implementing the ICF in the field of neurorehabilitation, it is important to anchor essential skills in the basic training of healthcare professionals. The Institute of Physiotherapy at FH JOANNEUM in Graz/Austria developed a concept to help students learn the necessary skills for implementing the ICF in a structured way. In the area of neurorehabilitation, we linked the ICF model with the Clinical Reasoning Model (CR). Competences are acquired over six semesters. Besides the general topics relating to the ICF (such as history, intention, and language) and CR that are taught in the first year, we focus in the later semesters explicitly on transferring these skills to neurorehabilitation. Therefore, we use interprofessional group work and problem-based courses as essential didactic elements for this transfer of skills. This article aims to show how the ICF could be implemented in Bachelor's degree programs for physiotherapy as well as in other healthcare programs. The authors’ experiences are described and some best practice examples when working with the ICF in this field are given.

## Introduction

The International Classification of Function, Disability, and Health (ICF) is known to be a valuable classification model in interprofessional neurorehabilitation, as it can lead to more patient-centered and self-determined treatment (1). Nevertheless, the possibilities of the ICF are still only partially utilized in both inpatient and outpatient settings due to a lack of knowledge about the possibilities and content of the ICF (2). To counteract this lack and to acquire the competencies to implement the ICF in the field of neurorehabilitation, it is important to anchor essential skills in the basic training of healthcare professionals.

In Austrian Bachelor’s programs for physiotherapy, ICF holds much importance. One notable aspect is the comprehensive integration of this model into various stages of the physiotherapy process. This integration encompasses history-taking, hypothesis generation, physiotherapeutic diagnosis, therapy planning, and assessment of therapy goals (3). This structured approach is commonly recognized as clinical reasoning, a cognitive process essential for effective decision-making in clinical settings. Clinical reasoning involves critical thinking within a clinical context, aimed at gaining a holistic understanding of a patient's condition and needs (4). Even though the implementation of the ICF in basic physiotherapy training is not new and has been an integral part of the curriculum for many years, the implementation of theoretical aspects in everyday practice proved to be particularly challenging. For this reason, FH JOANNEUM places particular emphasis on implementing the comprehensive ICF components from theory into practice in a joint decision-making process involving internal and external lecturers as well as internship supervisors. These parties were therefore also involved in the development of the teaching tools and clinical reasoning processes, which to our knowledge is a unique feature in this context.

In Austria, there is a strong recommendation for the inclusion of learning outcomes (LOs) related to the application of the ICF in Bachelor’s programs for physiotherapy, particularly concerning neurological patients (5). This emphasis underscores the significance of integrating the ICF framework into the curriculum to enhance students’ competence in assessing and treating neurological conditions effectively. Consequently, this integration is deemed an indispensable component of the Bachelor's program in physiotherapy. In response to this requirement, the Institute of Physiotherapy at FH JOANNEUM in Graz, Austria, devised a tailored strategy, in the period from 2012 to 2015, to ensure that students acquire the necessary competencies for applying the ICF framework within a structured approach.

Clinical reasoning involves healthcare professionals and patients collaboratively analyzing clinical issues to guide evidence-based practice. The abovementioned holistic approach encompasses assessing physical dysfunctions, environmental factors, and personal aspects in neurologically affected patients. This comprehensive evaluation extends beyond dysfunction to activity and participation levels, fostering multidisciplinary communication and care and so fits within the ICF and vice versa (6).

This article aims to demonstrate the practical implementation of the International Classification of Functioning, Disability, and Health (ICF) within Bachelor's degree programs for physiotherapy and other healthcare disciplines. It focuses on the implementation of the ICF concept in the specific field of physiotherapy in neurology. First, we illustrate how and why students learn to apply the ICF and CR models for neurological patients, with particular emphasis on hypothesis generation and evaluation. Then we outline the learning outcomes students should achieve when applying the ICF structures in neurology. For this purpose, we provide a brief insight into how the history, idea, and nomenclature of the ICF are introduced in the initial semesters so that foundational knowledge can be drawn upon when students learn about neurological patients. Finally, the methods implemented in the courses are described.

Drawing from the author's experiences, the article offers insights into effective strategies for integrating the ICF into academic curricula. In addition, it provides best practice examples of utilizing the ICF framework within the realm of physiotherapy education and practice.

## CR and ICF in physiotherapy for neurological patients

Clinical reasoning can be defined as a reflective process of examination and analysis carried out by healthcare professionals in collaboration with patients to understand them, their context, and their clinical problem(s) to enable evidence-based practice (4). In terms of the biopsychosocial framework (7), a comprehensive clinical thought process also attempts to capture all levels of the ICF (8) and thus provides a holistic approach when examining and treating neurologically affected patients.

This holistic approach therefore includes the examination and analysis processes for general physical health or dysfunctions, such as cardiovascular conditions or the clinical consequences of upper motor neuron lesions (including spasticity, weakness, hyperreflexia), but also the corresponding examination of environmental and personal factors. These include living conditions, like barriers due to stairs, connection to the healthcare network due to mobility restrictions, but also personal aspects such as cognitive impairments or neuropsychological disorders (aphasia, neglect, depression).

The critical thinking process in the context of clinical action leads to a comprehensive assessment of the situation not only at the level of dysfunction but above all at the level of activity and participation. Furthermore, this holistic approach facilitates open communication with all those involved with the aim of a comprehensive multiprofessional approach, i.e., including professional groups such as psychologists, social workers, and, of course, nursing staff.

Considering all these aspects it seems more than reasonable that students in the Bachelor’s program learn to apply the CR model to neurological patients and to integrate the ICF model into the CR model. At FH JOANNEUM, hypothesis generation related to the ICF is pivotal.

As part of the clinical reasoning process, our students learn to form so-called hypothesis categories (4) during their basic training already in the first year. Even though these hypothesis categories originated in the treatment of musculoskeletal problems (9), they can be transferred to all clinical areas. Table 1 presents the hypothesis categories we currently use as a guide and gives examples of clinical characteristics for neurological patients.

**Table 1:** Hypothesis categories and clinical examples.

Hypothesis categories	Clinical features
Activity and participation capability and restriction	“Walking a distance of 150 m and back with crutches to manage a shopping tour three times a week”
Patient perspectives on their experiences and social influences (psychosocial status)	“Lack of motivation to build up aerobic capacity after stroke”
	“Lack of acknowledging the current disability situation and denying help from relatives”
	“Mood disturbances following a stroke due to realizing disabilities”
Pain type	“Neuropathic pain due to shoulder-arm syndrome”
	“Nociceptive pain due to spasticity and adaptive phenomenon”
Source of symptoms	“Arteria cerebri media-associated stroke and its associated brain regions leading to an upper motor neuron syndrome (UMNS)”
Pathology	“Brain regions hypoxic situation due to thrombosis”
Impairments in body function or structure	“UMNS-associated dysfunctions due to spasticity in the left elbow region”
	“Weakness/paresis of quadriceps muscle left”
	“Loss of sensory dysfunction, facial region right”
Contributing factors	“High blood pressure”
	“Diabetes mellitus type 1”
	“Hip arthroplasty 2 years ago on the contralateral side”
Precautions and contraindications to physical examination and treatment	“Anticoagulation”
	“Neglect”
	“Heart insufficiency”
Management and treatments	“Top down model of treatment because of the good neuropsychological state and the fast remission”
	“Teaching and guiding relatives in transfer”
	“Multiprofessional approach because of neuropsychological contribution”
Prognosis	“Depending on resources—barrier profile regarding ICF aspect in personal and system contribution”

The physiotherapy assessment aims to provide adequate information for the clinical reasoning categories. The data collected from the medical history and physical examination can then be used to create a comprehensive patient-centered assessment and plan and help implement appropriate individual therapy (10). The functional, activity, and participation levels are reflected in the hypothesis categories, and personal and environmental considerations are also adequately recorded from the outset in the contributing factors or psychosocial factors.

## Learning outcomes

When implementing the ICF into the Bachelor’s program for physiotherapy, our curriculum coordinators opted for a strong orientation toward the consensus paper of the Austrian University Network Physiotherapy in Neurology (ÖHPN) (5). Table 2 provides the LOs that explicitly refer to the ICF.

**Table 2:** The learning outcomes of the ÖHPN for the expert role, with explicitly mentioning the ICF or the ICF domains.

Part of the physiotherapy process	Learning outcomes The graduates…
Examination	…Plan to conduct the examination, starting from the level of activity and participation, leading to the level of structure and function.
* *	…Identify deficits and resources across all areas of activity and participation such as mobility, communication, and self-care
* *	…Utilize specific assessment tools at the structural and functional levels, as well as at the activity and participation levels, for differentiated problem identification and evaluation.
Making a physiotherapeutic diagnosis	…Establish in the physiotherapeutic diagnosis, connections between the collected results and categorize them in relation to all ICF levels, prioritizing the limiting factors while considering the complexity of neurological disorders for all ICF levels.
Formulation of goals	…Initially formulate goals at the level of activity and participation, as well as problem-based goals at the functional and structural levels.
* *	…Align goal formulation with the ICF structure
Planning and implementation of interventions	…Apply manual techniques (manual therapy, soft tissue techniques, sensory stimulation, neurodynamics) at the level of body structure and function in patients with neurological conditions to enable or facilitate activities and functions.
	…Support resource-oriented learning of patients
Re-examination	…Apply qualitative and quantitative reassessments (e.g., symptom-specific assessments) at all ICF levels, considering neurological and neuropsychological symptoms and environmental contextual factors.
* *	…Evaluate hypotheses based on the examination results regarding all activity areas (mobility, communication, self-care).

The specific implementation of the LOs at FH JOANNEUM focuses on examination, physical therapist (PT) diagnosis, goal formulation, treatment strategies, and reassessment. In the examination domain, third-semester students concentrate on the activity levels of the ICF relevant to neurology. They learn and understand how to identify and consider these levels while taking a patient’s history and during the examination. The relevant activity levels include those mentioned in ICF Chapters 3 (communication), 4 (mobility), and 5 (self-care).

Students also learn to implement and assess contextual factors while taking a patient's history, identifying these factors as resources or barriers to the patient's presenting problem. These components are essential for critically evaluating the patient's prognosis, which is an integrative part of the clinical reasoning process. Following the abovementioned hypothesis formation process, they subsequently learn to assess and examine the patient's limitations at the functional and structural levels of the ICF. These limitations are identified during history-taking and documented in a body chart. Physiotherapy students are instructed to examine and to use assessments across all ICF levels. The selection of the assessments is based on an unpublished Delphi process of the ÖHPN inquiring about which assessments should be taught in the Bachelor’s program for physiotherapists in Austria and are closely aligned with internship providers` requirement. The students are also introduced to ICF Core Sets, which serve as guidelines for identifying relevant activities to assess specific neurological conditions.

After hypothesis evaluation, during the examination, students are facilitated to establish a “Physiotherapeutic Diagnosis” prioritizing and describing the individually relevant limitations at the examined and evaluated activity level. Further, they are encouraged to identify the limitations at the functional and structural levels responsible for the patient's main problem.

The ICF domains serve as guidance for students in establishing common goals with the patient. In neurology, due to certain communication limitations (aphasia, dysarthria, comatose situations) of patients, close cooperation with the nursing staff and patient's relatives is required to enable a joint process of goal setting. It is therefore important that students learn to communicate with all involved people. Expanding upon the physiotherapy diagnosis, students are instructed in developing comprehensive treatment plans. This involves learning interventions that target activity and functional/structural levels, incorporating a blend of hands-on and hands-off techniques.

Students learn about qualitative and quantitative assessments for reassessment and can carry them out at both functional and activity levels.

## Methodic and didactic concepts and tools

During the six semesters, students acquire the competence and skills to apply the ICF to all clinical areas. At this point, we will concentrate on describing the three most important didactic considerations and methods in connection with the CR process in neurological patients.

## Development from novices to experts

Students do not only learn about the goals and nomenclature of the ICF, but they also learn how to use a process document in the first two semesters that we have developed as a learning tool, especially for this purpose.

As in the first two semesters students learn to reflect on this process in the musculoskeletal (MSK) area, in the third and fourth semesters, neurological clinical patterns, diagnoses, and patient cases are taught from a theoretical and practical point of view and so the critical thinking process is easier to reflect and adapt. Following the mentioned theoretical and practical content, students need to learn and practice the neurologically relevant hypotheses supported by the ICF. For example, they learn to develop hypotheses on neurological disorders at the activity and functional levels using audio and video recordings.

By documenting the described thinking process more linearly it seems easier for the students to follow a structured guideline in the subjective and objective examination. This process leads from novice structured thinking to more routine expertise thinking and acting by learning more clinical patterns and developing more experience in the clinical field *per se* (11).

During the subjective examinations, students are encouraged to explicitly focus on activities and participation issues that are related to the objective and subjective main problems. First, through an online pre-reading assignment using the ICF catalog (Chapters d3–d6), they gain an overview of activity limitations that might affect neurological patients. Then, in class, they learn with the help of a table (Supplementary Material S1, page 2) to relate specific activities to the contextual factors based on simple case studies. Here they already reflect on the barriers that might occur and hinder performing explicit tasks. One example for neurological patients from the ICF activity area of conscious sensory perception or communication is maintaining eye contact. Students should also document whether the activity is better possible on one side than on the other or whether the activity is also possible with a dual task requirement. Another example would be whether or how much assistance is required for personal hygiene.

When forming hypotheses regarding functional and structural levels, students are tasked with considering constraints within the categories of “Source of Symptoms,” “Pathology,” and “Impairments in Body Function.” These limitations, particularly relevant to neurological patients, may manifest across various functional domains.

In prognostic hypothesis formation, students are encouraged to integrate contextual factors they have gathered. It is crucial to account for the environmental context in which participation takes place, thereby emphasizing the importance of performance and potential or necessary support. In the context of the ICF, the restoration of activities and participation is associated with a variety of considerations. This ranges from determining the intensity of training to the selection of suitable aids. It is now evident that a more refined and precise clinical thinking process develops through training in these linear processes and reflecting on them not only during teaching sessions but also after practical experience in healthcare facilities. This progression is recognized and reinforced by teachers and is covered in various learning modules over the semesters.

## Interprofessional exchange in neurorehabilitation

Concerning the LO of the ÖHPN (5) in the role profile of the team worker, we established an interprofessional course. The aim was to enable students to engage in interprofessional discourse using the ICF as a common language.

For this purpose, an interdisciplinary teaching event is conducted when first students from physio and occupational therapy engage in a joint exchange to align, supplement, and discuss knowledge about ICF content. In the first phase, organized in small groups, they should discuss their understanding regarding the goals and objectives of the ICF, share any relevant experiences from internships, and explore opportunities related to the ICF within the context of healthcare. Then these results will be presented and discussed in the plenum.

In the next phase, mixed small groups discuss actual neurological cases, aiming to develop a collaborative case review process. In addition, students work together to devise therapy plans outlining the capabilities of each professional group involved.

During the discussion and with the help of a role-play, students need to learn how to argue for prioritizing therapy goals or incorporating specific contextual factors by using the ICF terminology.

## Problem-based learning

As recommended (12), we use Problem-Based Learning (PBL) (13) as a learning tool to integrate the ICF into the CR process using real patient cases. In this setting, students learn both communication skills and applying the ICF in the clinical reasoning process for neurological patients in semesters 3–6.

The three PBL sessions occur at the end of each semester. During these sessions, students engage in simulations to practice taking patient histories and with the aid of the abovementioned process, document (Supplementary Material S1) the assessment of neurological patients according to the ICF categories. Therefore, goals and treatments for both, the activity level and the functional level, must be found. Through simulation demonstrations, they refine their ability to integrate ICF terminology into client-oriented language. In the final step, they simulate planning and conducting workshops with neurological clients, implementing interventions at both, the activity and functional levels. A significant part of the debriefing focuses on feedback on the implementation of the ICF throughout the process.

## Discussion

Over the years, it has become evident that understanding the background, goals, and applications of the ICF is necessary to better integrate it into the clinical context and ensure patient-centered treatment (2, 14). Our contribution demonstrates one way of integrating the ICF already into undergraduate education.

Lexell and Brogårdh (15) describe assessment, goal setting, intervention, and outcome measurement as essential stages of neurorehabilitation. The ICF can be utilized in all these areas. The physiotherapeutic process in neurorehabilitation encompasses these exact areas in the CR process. We aim to enable students to acquire competence in the ICF and CR through the mentioned procedures and methods. As literature on evaluation methods for clinical reasoning processes that include ICF components is scarce, we propose a mix method of reflection processes, the necessity of feedback by internal teachers, and the application in problem-based learning classes and practical internships (16).

The functional descriptions across all levels of the ICF are essential for interprofessional exchange and collaborative goal setting (1). However, for the physiotherapeutic process, these formulations often are not sufficient. More explicit descriptions are needed, e.g., to plan a patient-centered movement analysis (see Table 1).

Physical therapy students at FH JOANNEUM learn about the possibility of using ICF Core Sets for neurological disorders, enabling them to use these sets to identify and monitor functional impairments at various levels. However, for physiotherapeutic treatment planning, an explicit analysis of the patient's movement behavior is additionally required. For example, to plan training aimed at improving gait pattern functions (GPF) (b770), it is essential to precisely determine the timing, extent, localization, and modifiability of the GPF.

As described, we base our approach on the consensus paper of the ÖPHN (5). There is one further LO when applying the ICF. In their role as health promoters, students should learn to use motivating, health-promoting measures to change behavior and relationships. Moreover, patients with chronic neurological conditions often belong to the group of individuals with low health literacy (17). Our students learn to assess and promote health literacy in such a way that they can help patients utilize health information, particularly in the domains of participation and activity autonomously.

Written and oral exams are utilized to assess the attainment of learning objectives. However, there is currently no available data regarding the satisfaction of graduates’ employers with their proficiency in the International Classification of Functioning, Disability, and Health (ICF). To address this gap, a qualitative survey of institutions employing graduates is being contemplated.

Incorporating the ICF into Bachelor’s programs for healthcare professions is instrumental in preparing graduates for the workforce. Not only does it improve therapy planning and executing skills in their own professional field, but it also fosters interprofessional communication and collaborative practices (18), for which there is a great need in the field of neurorehabilitation (19). To further enrich the training experience, we are broadening the program to include occupational therapy and nursing students in interprofessional training. Moreover, we plan to integrate problem-based learning phases involving students and instructors from European partner universities in the future.

## Data Availability

The original contributions presented in the study are included in the article/Supplementary Material, further inquiries can be directed to the corresponding author.

## References

[1] Leonardi M Fheodoroff K. “Goal setting with ICF (International Classification of Functioning, Disability and Health) and multidisciplinary team approach in stroke rehabilitation”. In: Platz T, editor. Clinical Pathways in Stroke Rehabilitation: Evidence-Based Clinical Practice Recommendations. Cham: Springer International Publishing (2021). p. 35–56. 10.1007/978-3-030-58505-1_3 36315695

[2] Aftenberger H Schwarze G Salchinger B Rother A. The International Classification of Functioning, Disability and Health (ICF) in neurorehabilitation in Austria. Comparison of the healthprofessions occupational therapy, speech-and language therapy and physiotherapy/Die Internationale Klassifikation der Funktionsfähigkeit, Behinderung und Gesundheit in der Neurorehabilitation in Österreich. Vergleich der Berufsgruppen Ergotherapie, Logopädie und Physiotherapie. Int J Health Prof. (2017) 4(2):137–46. 10.1515/ijhp-2017-0024

[3] Eckler U Gödl-Purrer B Hurkmans E Igelsböck E Wiederin C. Die Physiotherapeutin/Der Physiotherapeut. Vienna: Kompetenzprofil (2017). Available online at: https://www.physioaustria.at/sites/default/files/collection_files/phy_kompetenzprofil_deutsch_fin_072017.pdf.

[4] Jones MA Rivett DA. Clinical Reasoning in Musculoskeletal Practice (2. Aufl.). London: Elsevier (2019).

[5] Lotter K Kidritsch A Aftenberger H Mayrhofer G Polanz K Riedl T Learning outcomes physiotherapy in neurology—a structured consensus finding of the Austrian University Network Physiotherapy in Neurology (ÖHPN)/learning outcomes physiotherapie in der neurologie—eine strukturierte konsensfindung des österreichischen hochschulnetzwerkes physiotherapie in der neurologie (ÖHPN). Int J Health Prof. (2020) 7(1):66–83. 10.2478/ijhp-2020-0007

[6] Maart S Sykes C. Expanding on the use of the international classification of functioning, disability and health: examples and resources. S Afr J Physiother. (2022) 78(1):1614. 10.4102/sajp.v78i1.1614 36092967 PMC9453191

[7] Engel GL. The need for a new medical model: a challenge for biomedicine. Science. (1977) 196(4286):129–36. 10.1126/science.847460 847460

[8] Ustün TB Chatterji S Bickenbach J Kostanjsek N Schneider M. The international classification of functioning, disability and health: a new tool for understanding disability and health. Disabil Rehabil. (2003) 25(11–12):565–71. 10.1080/0963828031000137063 12959329

[9] Jones MA. Clinical reasoning in manual therapy. Phys Ther. (1992) 72(12):875–84. 10.1093/ptj/72.12.875 1454863

[10] Luomajoki H Schesser R. Schmerzmechanismen und clinical reasoning. Der Schmerzpatient. (2018) 1(01):7–18. 10.1055/s-0043-122097

[11] Richards JB Hayes MM Schwartzstein RM. Teaching clinical reasoning and critical thinking. Chest. (2020) 158(4):1617–28. 10.1016/j.chest.2020.05.525 32450242

[12] World Health Organization. How to Use the ICF: A Practical Manual for Using the International Classification of Functioning, Disability and Health (ICF). Exposure Draft for Comment. Geneva: WHO (2013). Available online at: https://www.who.int/docs/default-source/classification/icf/drafticfpracticalmanual2.pdf?sfvrsn=8a214b01_4.

[13] Schmidt HG. Problem-based learning: rationale and description. Med Educ. (1983) 17(1):11–6. 10.1111/j.1365-2923.1983.tb01086.x 6823214

[14] Leonardi M Lee H Kostanjsek N Fornari A Raggi A Martinuzzi A 20 Years of ICF—International Classification of Functioning, Disability and Health: uses and applications around the world. Int J Environ Res Public Health. (2022) 19(18):11321. 10.3390/ijerph191811321 36141593 PMC9517056

[15] Lexell J Brogårdh C. The use of ICF in the neurorehabilitation process. NeuroRehabilitation. (2015) 36(1):5–9. 10.3233/NRE-141184 25547759

[16] Lennon O Phelan D Wallace D King J Barrett T. “The more you did, the more it made sense”: problem-based learning to improve early evidence-based practice in an undergraduate physiotherapy professional programme. Physiother Res Int. (2019) 24(3):e1774. 10.1002/pri.1774 30994262

[17] Byrne D. Understanding and mitigating low health literacy. Nurs Stand. (2022) 37(10):27–34. 10.7748/ns.2022.e11875 35856242

[18] Moran M Bickford J Barradell S Scholten I. Embedding the international classification of functioning, disability and health in health professions curricula to enable interprofessional education and collaborative practice. J Med Educ Curric Dev. (2020) 7:2382120520933855. 10.1177/2382120520933855 32944651 PMC7466904

[19] Schwab SM Dugan S Riley MA. Reciprocal influence of mobility and speech-language: advancing physical therapy and speech therapy cotreatment and collaboration for adults with neurological conditions. Phys Ther. (2021) 101(11):pzab196. 10.1093/ptj/pzab196 34403483 PMC8801003

