# Supplementary material for: ICF in Bachelor degree programs—the implementation of the ICF in the clinical reasoning process of physical therapists for neurological patients—optimizing the health curriculum for comprehensive patient care
Source: Front Rehabil Sci. 2024 Aug 9;5:1412163. doi: 10.3389/fresc.2024.1412163 (PMC11341536; doi:10.3389/fresc.2024.1412163)
Supplement: Supplementary file 1 [file Datasheet1.pdf]

Suppliment 1: Process document

Studierendenkennzahl_ Therapiesetting:	Alter, Geschlecht der Pat.:
Datum der Befundaufnahme:	Therapieanordnung:
Zuweisungsdiagnose:	Zusatzerkrankungen / Medikamente / Zusatzinfo:

1. ANAMNESE

1.1 Subjektives Hauptproblem:	1.2 Subjektives Ziel:

1.3 Geschichte der Zeichen und Symptome

1.4 Zeichen und Symptome auf ICF-Ebene Struktur – Body Chart:

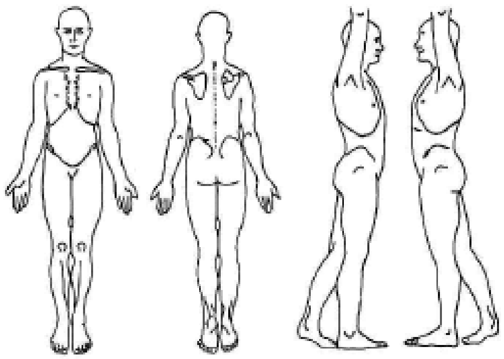

Zusammenhang der Zeichen und Symptome:

NRS / VAS

0 ———————————————— 10

1.5 Sozialanamnese:

Personenbezogene Kontextfaktoren im Sinne von Förderfaktoren (+) /Barrieren (-)	umweltbezogene Kontextfaktoren Sinne von Förderfaktoren (+) /Barrieren (-)

1.6 Zeichen und Symptome auf ICF-Ebene Aktivität/Partizipation

**Aktivität / Partizipation**	**Kontext** (wie viel Hilfe wobei? Wer? Wie?)

NRS → Aktivität

0 ———————————————— 10

Spezielle Fragen:

2. PLANUNG DER UNTERSUCHUNG

2.1 Hypothesen: (Fähigkeiten & Einschränkungen in Aktivität und Partizipation; Quelle der Symptome; Pathologie; Schmerz; Einschränkungen in Struktur und Funktion; beitragende Faktoren; psycho-soziale Status; Vorsicht/Kontraindikationen/red flags; Prognose; Management)

2.2 Planung der Untersuchung zur Verifizierung der Hypothesen

Belastbarkeit in Bezug auf die durchzuführenden Untersuchungen:

Untersuchungen / Messungen / Assessments:

Inspektion:

Palpation:

Untersuchung von Strukturen, Funktionen (Haltung und Bewegung):

Untersuchung von Aktivität und Partizipation:

3. **Überprüfung der Hypothesen durch die PHYSISCHE UNTERSUCHUNG / MESSUNGEN mittels** Inspektion, Palpation, Bewegungs- und Haltungsanalysen, (Zusatz)-Untersuchungen, Messungen, Assessments:

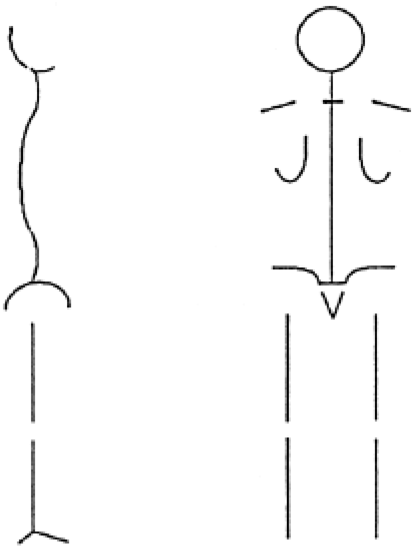

➔ inkl. Hypothesenevaluierung nach der Inspektion / Palpation:

➔ Hypothesenevaluierung nach der Bewegungs- und Haltungsanalyse:

➔ Hypothesenevaluierung nach der P/E, den Zusatzuntersuchungen und Assessments:

4. PHYSIOTHERAPEUTISCHE DIAGNOSE UND BEHANDLUNGSPLANUNG:

4.1 Zusammenführung der bestätigten Hypothesen (Fähigkeiten & Einschränkungen in Aktivität und Partizipation; Quelle der Symptome; Pathologie; Schmerz; Einschränkungen in Struktur und Funktion; beitragende Faktoren; psycho-sozialer Status; Vorsicht/Kontraindikationen/red flags; Prognose; Management)

4.2 Physiotherapeutische Diagnose:

4.3 Zielvereinbarung:

**...auf Aktivitäts- und/oder Partizipationsebene**
**Teilziele** (auf Aktivitäts-, Struktur- und Funktionsebene) – SMART
**Ressourcen** (Welche Ressourcen hat der/die Patient/in um die Ziele zu erreichen
**Prognose** (kritisches Einschätzen der Zielerreichbarkeit)

Behandlungsplanung

Teilziel	geplante Maßnahmen	Retest-Parameter

5. BEHANDLUNG und THERPIEVERLAUF

Dokumentation des Therapieverlaufs:

Datum	Hypothese(n) / Therapieeinheit	Behandlung und Therapieverlauf inkl. Retestergebnis

6. EVALUIERUNG und ENDBEFUND:

Überprüfung der Hypothesen und Zielerreichung

Therapieausblick
